# Preparation of Nickel Cobalt Sulfide Hollow Nanocolloids with Enhanced Electrochemical Property for Supercapacitors Application

**DOI:** 10.1038/srep25151

**Published:** 2016-04-26

**Authors:** Zhenhua Chen, Zhanghui Wan, Tiezhu Yang, Mengen Zhao, Xinyan Lv, Hao Wang, Xiuli Ren, Xifan Mei

**Affiliations:** 1 Jinzhou Medical University, Jinzhou, 121001, People’s Republic of China

## Abstract

Nanostructured functional materials with hollow interiors are considered to be good candidates for a variety of advanced applications. However, synthesis of uniform hollow nanocolloids with porous texture *via* wet chemistry method is still challenging. In this work, nickel cobalt precursors (NCP) in sub-micron sized spheres have been synthesized by a facile solvothermal method. The subsequent sulfurization process in hydrothermal system has changed the NCP to nickel cobalt sulfide (NCS) with porous texture. Importantly, the hollow interiors can be tuned through the sulfurization process by employing different dosage of sulfur source. The derived NCS products have been fabricated into supercapacitor electrodes and their electrochemical performances are measured and compared, where promising results were found for the next-generation high-performance electrochemical capacitors.

In the past few years, the nanostructures hollow nanocolloids have stimulated great interests in many advanced applications, such as drug delivery^1^,^2^, chemical sensors^3^,^4^, photocatalysis^5^ and energy storage^6^,^7^,^8^ due to their unique compositional and structural features in low mass density, high surface area and permeable shell structures^9^,^10^. As a result, well-defined hollow structures with controllable composition and morphology are attracting more and more attentions in terms of their broadening practical applications^11^. A great many efforts such as hard/soft template synthesis, sacrificial template method and template-free methodhave been made to prepare different kinds of hollow structures^12^. In some of the recent work, the importance of hollow materials in enhancing the electrochemical performances have been highlighted and emphasized, which may spark the further investigations in electrochemical devices by using different types of hollow materials^13^,^14^,^15^,^16^,^17^.

Supercapacitors are also known as electrochemical capacitors, which are considered to be one of the most important energy storage systems in the 21 century due to their excellent electrochemical features such as high power density, fast charge-discharge kinetics and very long cycle life compared to the battery counterparts^18^,^19^. Some recent reports have demonstrated their high reliability and good efficiency in practical applications, which witnessed the quick developments of this technology^20^. However, the electrochemical performances of the supercapacitor cells are highly depending on the types of electrode materials as well as their micro/nano morphologies, because most of the charge exchange and transfer occurred on the interface between the electrodes and electrolyte, which is the principal mechanism to determine the property of an electrochemical device^21^. Hence, it is desirable and vital to explore unique electrode materials with high electrochemical activity to fabricate high-performance supercapacitors. As key building components of the supercapacitor cells, various active materials have been studied to develop next-generation supercapacitors. Carbon materials and conducting polymers were commonly used for supercapacitor electrodes due to their low cost, ease of process ability, and controllable porosity^22^. But the relatively low specific capacitance of the carbon-based materials has prompted the material scientists to develop transitional-metal based materials and even hybrid materials^23^,^24^,^25^,^26^,^27^,^28^, which are able to deliver much higher capacitance owing to the Faradaic reactions involved in the charge-discharge process^29^. Chen *et al.* have prepared NiCo_2_S_4_ nanotube arrays directly on Ni foam, and their electrochemical results presented a high areal capacitance of 14.39 F cm^−2^ at a current density of 5 mA cm^−2 ^30. In another example, NiCo_2_S_4_ nanosheets were grown on conductive carbon substrate as electrode materials for supercapacitors. The 3D hybrid materials developed therein exhibited mesoporous texture with open framework, which have shown high specific capacitance with excellent capability^31^. In addition, a recent work by Cai *et al.* have tried to explore the NiCo_2_S_4_ with r-GO for enhanced electrochemical property owing to the excellent physical and chemical characteristics of graphene materials^32^. However, fabrication of pure NiCo_2_S_4_ nanocolloids with unique architecture and porous texture by facile and straightforward methods for improved capacitorperformances was less explored and discussed.

In this work, we have reported the synthesis of nickel cobalt sulfide (NCS) hollow spheres by a facile sulfurization of nickel cobalt precursors (NCP). NCP with sub-micronsized spheres were prepared *via* a facile solvothermal method, and the corresponding NCS products with porous texture were then obtained through a hydrothermal synthesis using sodium sulfide (Na_2_S) as sulfur source. By applying different dosage of Na_2_S, core-shell and complete hollow structures with high surface areas can be fabricated, respectively based on a sacrificial-template mechanism during the sulfurization process. Impressively, the as-derived NCS materials have exhibited high specific capacitance with excellent cycling performances when served as supercapacitor electrode materials.

## Experimental Section

### Synthesis of Ni-Co Precursor (NCP)

0.2 mmol of Ni(NO_3_)_2_ · 6 H_2_O and 0.4 mmol of Co(NO_3_)_2_ · 6H_2_O were dissolved in 25 mL of isopropanol (IPA) under magnetic stirring for 10 min, followed by the addition of 1 mL of ethylene glycol (EG). 2 min later, the mixture was sealed in a Teflon-lined autoclave and heated at 180 °C for 6 h. After cooling down naturally, the product was rinsed thoroughly with DI water/ethanol several times and collected by centrifugation followed by drying at 60 °C in an air-flow oven.

### Synthesis of Nickel Cobalt Sulfide (NCS)

30 mg of the as-prepared NCP was dispersed into 30 mL of ethanol by ultrasonication for 10 min, followed by the addition of Na_2_S. After hand-shaking for 5 min, the mixture was sealed in a Teflon-lined autoclave and then heated at 120 °C for 8 h. The reaction was allowed to cool down to room temperature naturally, and the black product was collected by the rinse-centrifugation process with DI water and ethanol several times. The obtained product was thoroughly dried at 60 °C in vacuum for further characterization. The sample NCS-1 and NCS-2 were prepared with 60 and 120 mg of Na_2_S, respectively.

### Material Characterizations

All the samples were characterized by field-emission scanning electron microscopy (FESEM, Hitachi, S-4800) operating at 15 kV equipped with an energy dispersive X-ray spectroscopy (EDX), transmission electron microscopy (TEM, FEI, Tecnai G2 F20 STwin, USA) with an accelerating voltage of 200 kV and X-ray diffraction (XRD, Shimadzu, X-Lab 6000 Diffractometer, Cu Ka, λ = 1.5406 Å). The texture properties of the relevant samples were carried out at 77 K with a Quantachrome NOVA-3000 system. The Brunauer-Emmett-Teller (BET) surface area was calculated using adsorption data in a relative pressure ranging from 0.05–0.3. The pore size distribution was estimated using the desorption isotherm based on the Barrett-Joyner-Halenda (BJH) method.

### Electrochemical Measurements

The supercapacitor electrodes were fabricated by mixing the active materials with carbon black and polyvinylidene difuoride (PVDF) at a weight ratio of 8:1:1. After thorough mixing, the slurry was pressed onto Ni foam and was dried at 60 °C in vacuum for 12 h. The electrochemical tests were performed with a CHI 660D electrochemical workstation in an aqueous KOH electrolyte (2 M) with a three-electrode cell where Pt foil served as the counter electrode and a standard calomel electrode (SCE) as the reference electrode. The EIS analysis for both of the two samples were carried out in the frequency range from 0.01 to 100 KHz at open circuit potential (0 V) with 5 mV amplitude.

## Results and Discussion

**Figure 1:**
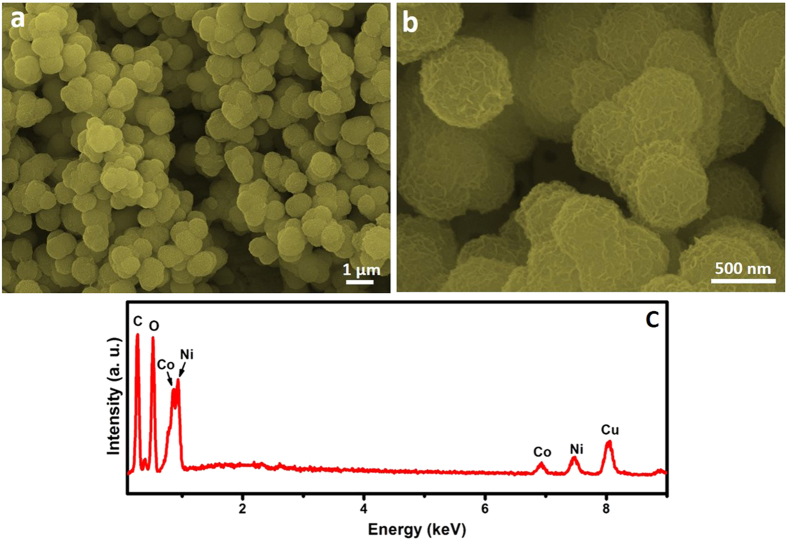
FESEM images (**a,b**) and EDX pattern (**c**) of the as-prepared Ni-Co precursor.

The NCP particles in spheres are displayed in the FESEM images, shown in Fig. 1a. The particles are in sub-micron size with nanosheets as building subunits, which can be observed in a magnified FESEM image (Fig. 1b). The composition of the as-synthesized NCP is further analyzed by EDX (Fig. 1c), where the presence of Ni, Co, O can be confirmed. The peaks of C and Cu come from the organic solvent in the synthesis and SEM substrate (sample solution was dropped onto the Cu substrate and then dried for FESEM characterizations). It should be noted that the EDX is a rough technic for the confirmation of the elements in the samples, but may not be accurate enough for their molecular ratios.

**Figure 2:**
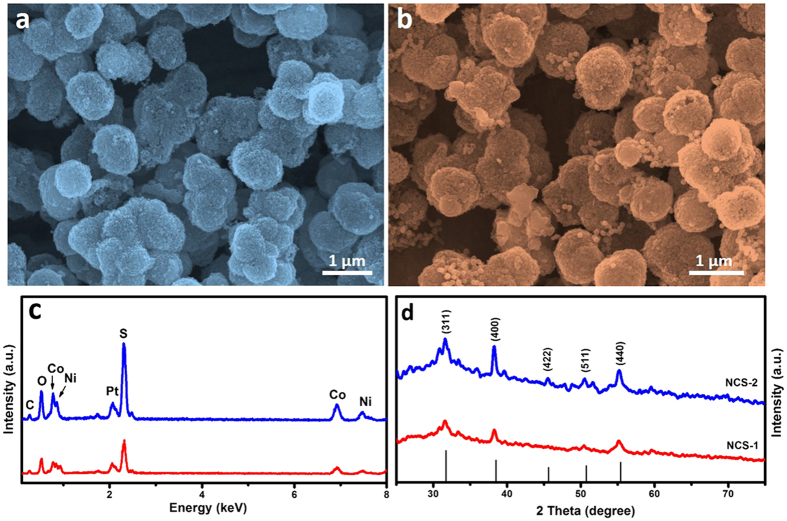
FESEM images (**a,b**), EDX (**c**) and XRD (**d**) patterns of the NCP derived NCS-1 (**a,c,d**) and NCS-2 (**b,c,d**) nanocolloids.

The as-prepared NCP were subsequently transformed into NCS samples *via* a facile hydrothermal method in the presence of Na_2_S and the results are shown in Fig. 2. By using different dosage of Na_2_S, core-shell structure of NCS-1 (Fig. 2a) and hollowed colloids of NCS-2 (Fig. 2b) can be obtained, respectively (confirmed by TEM later). It is interesting to see that the building nanosheets have been transformed into nanoparticles for both of the samples after hydrothermal sulfurization. The further EDX of both of the sulfurized samples shown in Fig. 2c indicates the formation of nickel cobalt sulfide materials due to the presence of the strong S peak as well as the Ni and Co peaks. In order to determine the crystal phases of the as-derived sulfide materials, XRD have been carried out and results are shown in Fig. 2d. All the indexed peaks can be attributed to the cubic NiCo_2_S_4_ (JCPDS card no. 20-0782)^33^. No additional signals from other impurities were detected, indicating the high purity of the derived NCS samples even though the peak intensities are not so strong.

**Figure 3:**
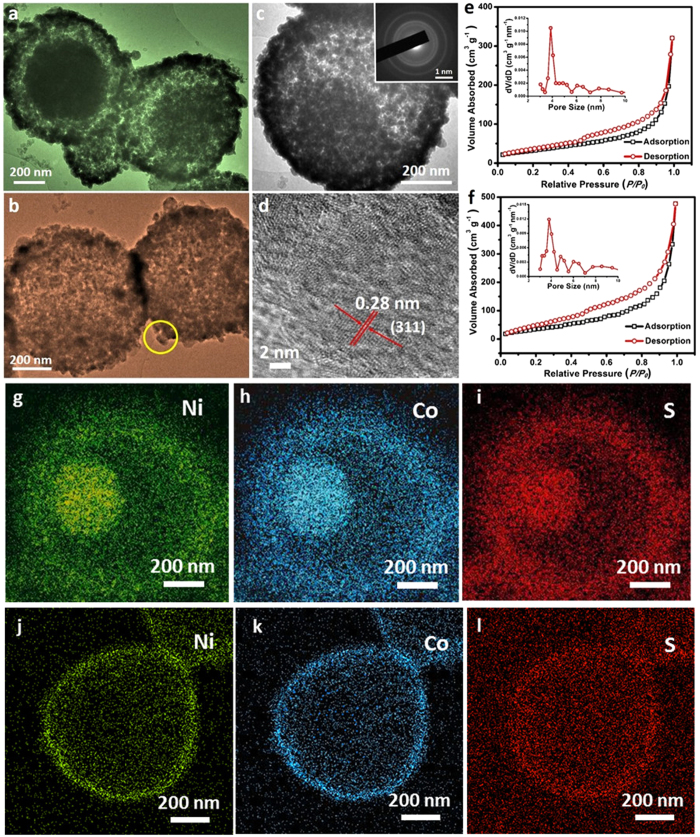
TEM images (**a–c**), HR-TEM (**d**), BET results (**e,f**) and element mapping (**g–l**) of the as-derived NCS-1 core-shell spheres (**a,c,e,g–i**) and NCS-2 hollow spheres (**b,d,f,j–l**).

The interior structures of the NCS particles are further examined by TEM, and images are shown in Fig. 3a–d. The unique core-shell structure of sample NCS-1 is revealed in Fig. 3a, where the gaps between the solid cores and shells can be observed clearly by the distinct contrast (Fig. 3c). The inset in Fig. 3c is a SAED pattern which shows the poly-crystallinity of sample NCS-1. The higher dosage of Na_2_S has leaded the NCP particles to a more porous structure with hollow voids (Fig. 3b, crystallite sizes estimated to be 24 nm, which are close to the 29 nm calculated by Scherrer equationbased on the XRD results), which infers that Ni and Co species have travelled from inner areas of the particles to external shells during the sulfurization process. Figure 3d shows a HR-TEM image selected from the area indicated by a yellow circle in Fig. 3c. A 0.28 nm of the lattice distance can be identified, corresponding to the crystal phase of (311)^34^. In terms of the large voids of the obtained particles, BET measurements were conducted to investigate the textural characteristics of the NCS samples and results are shown in Fig. 3e (NCS-1) and f (NCS-2).The obtained isotherms can be categorized as type IV with small hysteresis loops observed at a relative pressure of 0.4–0.9 for both of the samples. High specific surface areas (SSA) can be calculated to be 206 and 248 m^2^ g^−1^ for sample NCS-1 and 2, respectively, showing the high porosity of the two samples. The higher SSA of NCS-2 than that of NCS-1 may be due to the larger interior space created by the stronger sulfurization situation. It can be also concluded from the pore size distributions (insets in Fig. 3e,f) that both of the NCS samples have pores with diameter around 4 nm, revealing a mesoporous characteristic property that may be accessible for corresponding sized hydrated electrolyte ions^35^. In order to further demonstrate the structural features of the NCS samples, element mappings have been performed and the results are displayed in Fig. 3g–i.The elements of Ni, Co and S can be identified clearly for both of the samples, which confirmed the previous EDX results. In addition, the distribution of the elements is observed to be consistent with the TEM findings, verifying again the formation of the core-shell (NCS-1) and hollow (NCS-2) nanocolloids.

**Figure 4:**
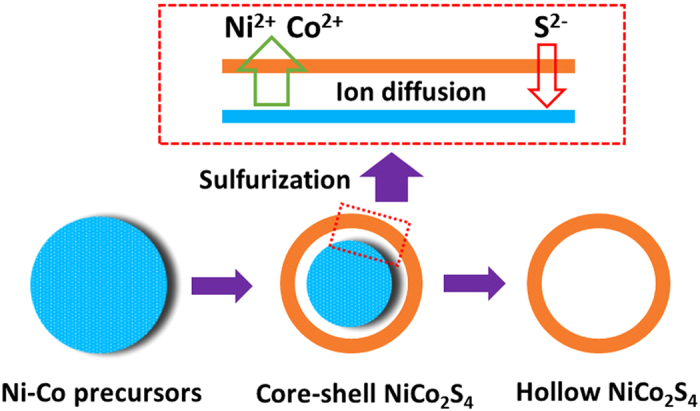
Schematic formation of the NiCo_2_S_4_ hollow spheres from Ni-Co precursor by a controlled sulfurization process.

Based on the results obtained, the ion diffusion can be employed to describe the formation process of the NCS samples (Fig. 4)^36^,^37^,^38^. During the hydrothermal sulfurization, S^2−^ released from Na_2_S will initially react with Ni/Co ions to generate a thin layer of Ni-Co sulfides, which has prevented the metal species further reacting with sulfur ions. Then the dissolution of NCP in hydrothermal system will release more Ni and Co ions, which will travel outwards to the pre-formed thin layer to form more NCS. As a result, core-shell structure and even complete hollow interiors are created finally based on the Kirkendall effect, which means that the hollow one (NCS-2) is the successive product of the core-shell (NCS-1) sample^39^.

**Figure 5:**
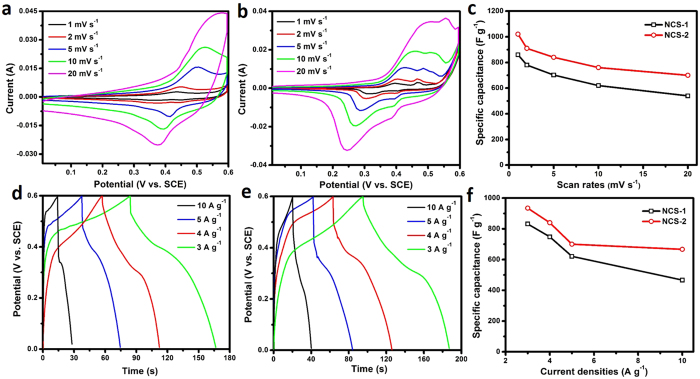
CV curves (**a,b**) conducted at different scan rates; specific capacitances calculated from different CV scan rates (**c**); charge-discharge curves obtained at different current densities (**d,e**) specific capacitances calculated from different discharge current densities (**f**) of the as-prepared NCS-1 (**a,c,d**,**f**) and NCS-2 (**b,c,e**,**f**).

In virtue of the unique structures and high surface areas, the as-derived NCS materials were used as electrodes for supercapacitors, and the electrochemical results are displayed in Fig. 5. The cyclic voltammetry (CV) results of both of the two samples are shown in Fig. 5a (NCS-1) and b (NCS-2), where pseudo-capacitive characteristics that different from the normal electric double-layer capacitance (a rectangular CV shape)^40^ can be seen obviously. The redox reactions involved during the charge and discharge process can be described as follows^38^,^41^,^42^:


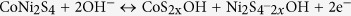


Based on the different CV scan rates at 1, 2, 5, 10 and 20 mV s^−1^, high specific capacitances of 860, 780, 702, 620 and 540 F g^−1^ can be calculated for sample NCS-1, while higher capacitance of 1021, 910, 840, 760 and 700 F g^−1^ can be calculated for sample NCS-2 with the same scan rates (Fig. 5c). The plateaus observed in the galvanostatic charge curves (Fig. 5d,e) have further manifested the anodic and cathodic process revealed in CV results. Corresponding capacitance calculations show that specific capacitances of 935, 840, 700 and 667 F g^−1^ can be delivered at current densities of 3, 4, 5 and 10 A g^−1^ for sample NCS-2, which exhibit higher values than those of sample NCS-1 (832, 748, 621, and 467 F g^−1^ are calculated) at the same current densities (Fig. 5f). The results reported herein are also comparable to some of the previous work. Zheng *et al.* reported the synthesis of NiCo_2_S_4_ hexagonal plates, which delivered a specific capacitance of 852 F g^−1 ^43. A very recent work by Shen *et al.* demonstrated a carbon foam supported NiCo_2_S_4_ nanosheets, which registered a capacitance of 877 F g^−1 ^44.

**Figure 6:**
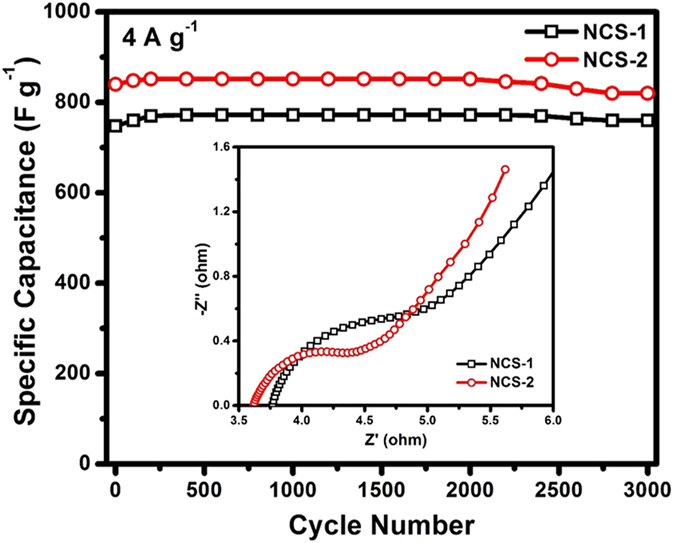
Cycling performances of the as-prepared NCS samples performed at a same current density of 4 A g^−1^ and the inset shows the Nyquist plots for both of the two samples.

The further investigations of cycling performance have verified the superiority of the as-prepared NCS materials (Fig. 6). At a current density of 4 A g^−1^, a high initial capacitance of 748 and 840 F g^−1^ are delivered for NCS-1 and NCS-2, respectively. It can also be seen from Fig. 5 that both of the two samples experienced a slight capacitance increase in the first 200 cycles, which may be due to the activation of the electrodes. Capacitances of 772 F g^−1^ and 852 F g^−1^ can be obtained for sample NCS-1 and 2, respectively after the electrodes are fully activated. The higher capacitance of NCS-2 is also revealed by the EIS analysis shown in the inset of Fig. 6. The first intersections of Nyquist plots on the Z’ axis in the high-frequency region reveal the high-frequency equivalent series resistance of capacitors (*R*s) which are contributed by the ohmic resistance of the electrolyte, the internal resistance of electrode materials, and contact resistance between electrodes and current collectors, while the semicircles crossing high and mid-frequency are attributed to the charge-transfer resistance^45^. The lower Rs together with the smaller radius of sample NCS-2 than NCS-1 can generally show a lower impedance and better electric conductivity, leading to a better electrochemical performance. After 3000 cycles, high capacitances of 760 F g^−1^ and 820 F g^−1^ can still be recovered for sample NCS-1 and 2, respectively, indicating good cycling retention of both of the two samples (98.4% for NCS-1 and 96.2% for NCS-2). It is interesting to note that sample NCS-1 shows a slightly higher capacitance retention than that of sample NCS-2. This could be attributed to the core-shell structure of NCS-1, where the core is additionally served as the mechanical support to stabilize the entire particle, favoring the preservation of the capacitance.

## Conclusion

In summary, we have reported the fabrication of functional NiCo_2_S_4_ nanocolliods by a facile wet chemistry method. The hollow interiors of the NCS particles can be readily tuned by adjusting the sulfurization extent during the hydrothermal process. The as-prepared NCS particles are uniform in size with high surface areas. In virtue of the unique structures and large surface areas, these NCS functional materials have exhibited high specific capacitance with excellent cycling stability as electrode materials, indicating their potential application in next-generation high-performance supercapacitors.

## Additional Information

**How to cite this article**: Chen, Z. *et al.* Preparation of Nickel Cobalt Sulfide Hollow Nanocolloids with Enhanced Electrochemical Property for Supercapacitors Application. *Sci. Rep.* **6**, 25151; doi: 10.1038/srep25151 (2016).
